# Colour of Medicines and Children’s Acceptability? A Systematic Literature Review of Children’s Perceptions about Colours of Oral Dosage Forms †

**DOI:** 10.3390/pharmaceutics15071992

**Published:** 2023-07-20

**Authors:** Elisa Alessandrini, Milena Gonakova, Hannah Batchelor, Sveinbjorn Gizurarson, Sonia Iurian, Sandra Klein, Daniel Schaufelberger, Roy Turner, Jennifer Walsh, Catherine Tuleu

**Affiliations:** 1 Department of Pharmaceutics, University College London School of Pharmacy, London WC1N 1AX, UK; milena.gonakova.21@ucl.ac.uk (M.G.); c.tuleu@ucl.ac.uk (C.T.); 2 Strathclyde Institute of Pharmacy and Biomedical Sciences, University of Strathclyde, Glasgow G4 0RE, UK; hannah.batchelor@strath.ac.uk; 3 Faculty of Pharmaceutical Sciences, University of Iceland, IS-107 Reykjavik, Iceland; sveinbj@hi.is; 4 Pharmacy Department, Kamuzu University of Health Sciences, Blantyre P.O. Box 360, Malawi; 5 Department of Pharmaceutical Technology and Biopharmacy, Iuliu Hatieganu University of Medicine and Pharmacy, 400012 Cluj-Napoca, Romania; iuriansonia@yahoo.com; 6 Department of Pharmacy, University of Greifswald, 17489 Greifswald, Germany; sandra.klein@uni-greifswald.de; 7 School of Medicine, Neurology, Johns Hopkins All Children’s Hospital, St. Petersburg, FL 33701, USA; dschauf1@jhmi.edu; 8 Idorsia Pharmaceuticals Ltd., 4123 Allschwil, Switzerland; roy.turner@idorsia.com; 9 Jenny Walsh Consulting Ltd., East Midlands Chamber, Nottingham NG1 1GF, UK; jenny@jennywalshconsulting.com

**Keywords:** colour, oral medicines, children, preferences

## Abstract

The colour of a product plays an important role in consumer experiences, and in the context of pharmaceutical products, this could potentially affect a patient’s expectations, behaviours, and adherence. Several studies have been conducted on adults, but little is known about children’s opinions on colours of medicines and to what extent medicines’ colour affects their acceptability. To address this gap, a systematic search in PubMed, Scopus, MEDLINE, and Web of Science was conducted. Two authors independently screened the titles, abstracts, and references of all articles and selected studies conducted on children (0–18 years old), assessing children’s preferences or opinions about colour of oral dosage forms as either a primary or secondary objective or as an anecdotal record. A total of 989 publications were identified and, after screening, 18 publications were included in the review. Red and pink were the most liked colours and there appeared to be a relationship between the colour of a medicine and expected taste/flavour. The review also highlighted a scarcity of information, usually collected as an anecdotal record. Several gaps in the current knowledge were underlined, emphasizing the need of patient-centred studies to understand if the use of certain colours can improve or worsen the acceptability of a paediatric medicine. This will help inform pharmaceutical manufacturers and regulators on the role and need of colours in children’s medicines beyond quality purposes.

## 1. Introduction

Colour plays an important role in consumer experiences, and in general, it is estimated that around 60% to 90% of a product assessment is usually based on colour alone [1]. This highlights the crucial role of colour and its ability to influence consumers’ perceptions about a product [2]. Thus, colour psychology is widely used in market research for the design of food and drink packaging, clothing, and much more [3].

In the pharmaceutical field, colouring agents are organoleptic excipients that are frequently added to pharmaceutical products [4], and their use is strictly regulated. In Europe, the use of colouring agents in medicinal products is governed by a specific directive (Directive 2009/35/EC of the European Parliament and of the Council) [5], and in the United States, the Federal Food, Drug, and Cosmetic Act (Chapter VII, Section 721) specifies that any colouring agent is subject to FDA approval before its use in drugs [6]. Likewise in Japan, the Ministry of Health, Labour, and Welfare and in Canada, the Government of Canada are responsible for defining and maintaining specifications [7]. These rules are to guarantee a safe and appropriate use of colouring agents.

Reasons for adding colour to a medicinal product are several: for improving brand recognition and to protect a product’s identity against generic competition [8,9], to help end-users recognise and differentiate medicines, including dose strengths, particularly when taking several medications [10]. Furthermore, colour can be associated with consumers’ perceptions affecting medicine efficacy via a placebo effect and triggering various emotional responses [11]. For example, red pills are generally considered stimulating, whereas blue pills are associated with a depressing or tranquilizing function [12,13,14]. Colour additives can also be used for formulation purposes, for example, to provide opacity to light sensitive active ingredients [15], or to match the visual appearance with the appropriate flavour of the formulation.

However, most of the research assessing the effects elicited by colours of medicines on the users has been limited to the adult population.

Children are even more sensitive than adults to sensory characteristics such as taste, size, or colour. It is known that children tend to like colours that match their own preferences [16,17], and these seem to lean towards bright colours particularly in young children, because these colours proved to evoke positive emotions (e.g., happiness) in children [16,17,18,19,20,21].

In recent years, there has been an increasing focus on making medicines for children more tailored for them and assessing a product acceptability has now become an integral part of the product development. Acceptability is defined as the overall ability and willingness of the patient and caregiver to use a medicinal product as intended. Acceptability is determined by the characteristics of the users and the product, and it is likely to have a significant impact on the patient’s adherence and, consequently, on safety and efficacy of the product [22]. The importance and need to study the acceptability of paediatric formulations was discussed in an EMA Reflection Paper [23] and endorsed in the latest EMA Paediatric guideline on pharmaceutical development of formulations for paediatric use [22], leading to more studies being conducted to evaluate product characteristics affecting acceptability and, in particular, palatability [24]. The appearance, including the colour, of a medicinal product is listed among the product characteristics influencing acceptability. However, knowledge about how children perceive colours of oral medicines and whether some colours could positively or negatively affect children’s acceptance for oral dosage forms, and to what extent, is lacking.

The use of a colouring agent in a paediatric product must be justified in terms of its safety in the proposed patient population, and potential benefits, including, for example, avoiding potential dosing errors and improved organoleptic acceptability [22]. Harnessing children’s views on their preferences could lead formulators to develop more acceptable medicines to them and enable informed decision-making regarding the need for or use for colours in paediatric products. Thus, the aim of this qualitative systematic review was to identify and collect available data about children’s views and preferences for colour of oral medicines from existing literature. These data will be used to assess to what extent this sensory attribute affects the overall medicine acceptability, and to identify any trend in terms of colour preferences among the paediatric population to understand which colours are preferable and which would need to be avoided to make a paediatric medicinal product more appealing. It will also be useful to identify any gaps in the current knowledge to be addressed by future studies.

This work was carried out by the conect4children (https://conect4children.org/, c4c (accessed on 8 June 2023)) Formulation Expert Group. c4c is a public–private consortium funded by the Innovative Medicines Initiative 2 to create a sustainable infrastructure promoting innovation in the design and conduct of paediatric clinical trials. The c4c Formulation Expert Group is one of the c4c methodology expert groups, and their focus is on formulation for children, providing advice on formulation aspects during children’s drug development. This study was carried out by the group for learning and providing new knowledge about how to further improve paediatric formulations, as this is important for the development of better medicines for children.

## 2. Materials and Methods

This systematic review was performed in accordance with the PRISMA guidelines of 2020 [25], and it was registered with the international Prospective Register of Systematic Reviews (PROSPERO), registration number CRD42022383533 (https://www.crd.york.ac.uk/prospero/display_record.php?RecordID=383533 (accessed on 30 May 2023)).

The SPIDER tool [26] was used to define the search strategy, as this was a qualitative review.

No restrictions were imposed in terms of type of study design to be included. However, due to the type of research question, it was foreseen that more qualitative rather than quantitative studies would have covered this topic.

The review included studies conducted in children aged between 0 and 18 years, and/or adult caregivers answering questions on behalf of their children. Children in the study could have been healthy and/or sick, and with or without any experience of taking oral medicines.

Since it was foreseen that not many studies collected information about colour perceptions in children, the evaluation of children’s preferences or opinions on colour of medicines could have been a primary or secondary outcome of the study, or an anecdotal record. To be included in the review, the study should have contained at least one mention about children’s perceptions or opinions on the colour of oral medicines.

No restrictions were placed in terms of when the study was published and the language of the study.

### 2.1. Search Strategy and Study Selection

The following databases were used: PubMed, MEDILINE (Ovid), Web of Science, and Scopus. The searches were conducted between August 2022 and November 2022. The search terms used for each database are provided in Appendix A. Reference lists and related literature of the articles collected from the databases were also screened.

The database records obtained from each search strategy were exported in an Excel file, and duplicates were removed manually.

Two reviewers (EA and MG) independently screened the title and abstract of articles obtained from the search and reference lists of selected articles against the inclusion criteria. Articles selected at this stage were further assessed for eligibility by screening the full text. All uncertain citations were included for full text review. Discrepancies in reviewers’ selection were resolved through consultation with a third review author.

### 2.2. Data Extraction

Data extraction was performed by one reviewer (EA). Relevant data were reported in a spreadsheet, and the following items were collected: year of the study, study design, whether colour was assessed as a primary aim or not, and methodology used to collect such information, topic assessed around colour, relevant results, dosage form and medication type, country where the study was conducted, details about participants (i.e., age, health status), and sample size.

The quality of each study was evaluated by using the Critical Appraisal Skills Programme (CASP) qualitative checklist [27]. Since the CASP checklist does not provide a scoring system, the scoring system adopted by Butler et al. [28] was used and adapted to define the level of quality for each study (see Table 1).

## 3. Results

### 3.1. Generality about the Studies

**Figure 1:**
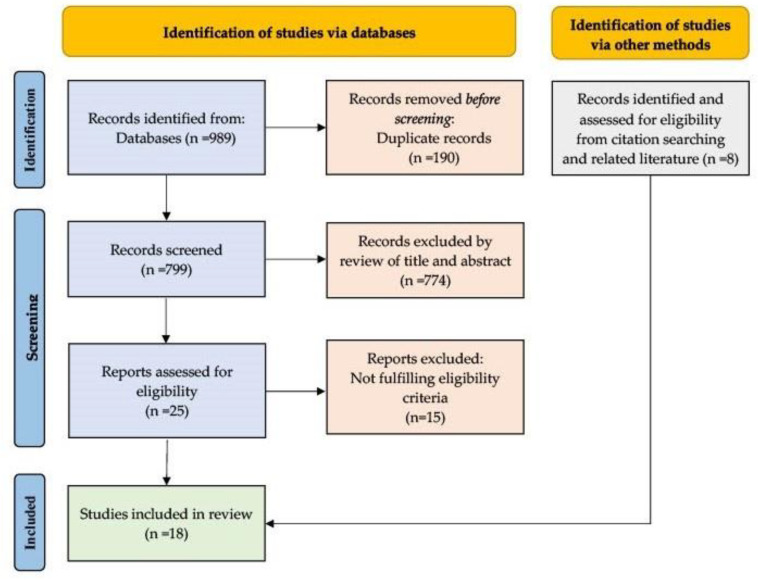
PRISMA 2020 flow diagram for new systematic reviews which included searches of databases, registries, and other sources [25].

A total of 989 publications were identified from the databases as potentially relevant. After the exclusion of duplicates and the screening process, 10 publications were deemed suitable for inclusion. Moreover, an additional eight publications were included following a manual screening of references and related literature. In the end, a total of 18 publications were included in the review (see Figure 1 and Table 2).

Most of the publications included were qualitative studies with the following study designs: surveys/questionnaires (n = 10), observational studies (n = 4), semi-structured interviews/focus groups (n = 3), and a prospective cross-over study (n = 1).

According to the CASP checklist and the adapted scoring system, 12/18 publications were scored as high-quality studies, 4/18 as moderate quality, and 2/18 as low-quality studies. This evaluation referred to the general aim of the study and did not take in consideration the methodology used to assess the colour of medicines. Thus, studies were not excluded based on their level of quality.

The publication year of the studies ranged from 1958 to 2022, with more studies being published in recent years, i.e., 12/18 publications were published in the last decade.

The age range of the children included in the studies varied considerably from one study to another, with some studies including children as young as one year old up to 18 years. Most of the studies (n = 14) were conducted entirely with children, two studies involved both children and caregivers, whereas two studies were entirely conducted in caregivers.

The sample size of the studies varied substantially, with the smallest study including 4 participants and the largest one including 842 participants.

Nine studies were performed on healthy children, six on children with a medical condition, e.g., chronically ill children, one study included both children with a condition and healthy children, and for two studies, this was not specified.

There were eleven studies conducted in Europe, of which eight were performed in the United Kingdom, five had been conducted in Asia, two in North America, two in Africa, and one in Oceania, as shown in Table 3. Some studies were conducted in more than one country.

In terms of dosage forms assessed, seven studies evaluated solid dosage forms, these included: tablets (n = 7), capsules (n = 2), granules (n = 1), chewable tablets (n = 1), minitablets (n = 2), 3D printed tablets (n = 2). There were five studies evaluating liquid dosage forms, two studies included both liquid and solid dosage forms, and four studies did not specify the type of dosage form assessed. Only six studies specified whether they were looking at a prescription or over the counter (OTC) medicine and five of them specified the medication class (antibiotics n = 2, antimalarial n = 1, analgesic n = 1, for metabolic disease n = 1). For the remaining 12 studies, this information was not available.

### 3.2. Information about the Colour of Medicines

Only five of the publications assessed the colour of medicines as a primary aim of their research, whereas most of the studies evaluated colour of medicines as a secondary aim or mentioned colour as an anecdotal record (n = 13).

To assess colour preferences or to collect information about colour among the children, several methodologies were applied (see Table 4). A physical evaluation of coloured medicines (tablets/liquids) or photographs of medicines (n = 4), the use of hedonic scales/acceptability preference questionnaires (n = 2), open or closed questions or a mix of the two (n = 2, n = 2, and n = 1, respectively), and a list of colours to choose from (n = 1) was made. However, for six studies, it was not clear or specified how information about colour was collected.

**Figure 2:**
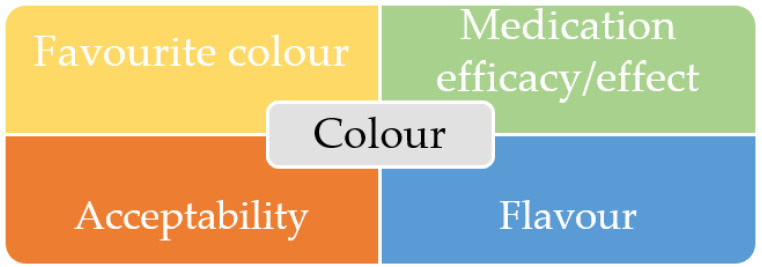
Topics assessed around colour of medicines in the studies retrieved.

The publications included in the review were classified in three subgroups according to the topic assessed around colour: (1) assessment of the most favourite colour (n = 9/18), (2) assessment of the relationship between colour of medicines and medication efficacy/effect (n = 4/18), and (3) effect of colour on acceptability (n = 5/18). Furthermore, the relationship between colour and flavour was assessed (see Figure 2).

### 3.3. Assessment of the Most Favourite Colour

Seven of the nine studies reporting information about children’s most favourite colour for oral medicines provided a colour ranking. However, several variations existed in the number and selection of colours used. Furthermore, without any rationale provided behind the colour choices in any of these studies, it was difficult to compare their outcomes, and there was a lack of well-designed prospective studies.

Red appeared to be the most favourite colour for both liquid and solid products. Red was selected as the first or second choice in 6/9 studies. Pink appeared to be the second most liked colour, with 3/9 studies reporting it as the most favourite colour and 2/9 studies as second choice. However, not all the studies providing a ranking included red or pink. Two recurring colours that were mentioned in all seven studies were blue and yellow, although these two colours were never among children’s primary choices, and yellow was always listed towards the end.

It was difficult to understand what children thought of other colours, as their position in the ranking changed among the studies. Orange seemed to have some interest being ranked as the first option in two studies. Interestingly, white never came first, it was usually towards the end of the list or in the middle. Other colours that were usually left towards the end of the ranking were brown and yellow, blue, black, and green, suggesting that children may not find medicines in these colours appealing.

The effect of gender on colour preferences was assessed by three studies. All three studies agreed that there seemed to be a “gender difference”, and that girls preferred pink medicines. However, these studies seemed to disagree about boys’ most favourite colour (blue/white/green).

It was not possible to draw any conclusion about cross-cultural and/or geographical differences in colour preferences, due to the small number of studies retrieved and lack of methodological alignment. Similarly, it was not possible to assess whether different preferences existed between different age groups, and between children with a chronic condition and healthy children.

### 3.4. Relationship between Colour and Medication Efficacy/Effect

Rather than looking at colour preferences, 4/18 studies assessed if the colour of a medicine affected drug efficacy or if children’s beliefs about a drug effect were affected by its colour. Two studies, both conducted in Asia (Malaysia and Indonesia), investigated the effect of colour on drug efficacy in children aged between 10 and 14 years. From one, it emerged that more than half of the children knew that efficacy was not related to the drug colour [37]; however, from the other study, it appeared that only 38% of the children were aware of that [43]. Whatley et al. [36] showed that most of the children were able to correctly identify bicoloured capsules as medicines compared to either white or pink tablets. White tablets were correctly identified only when the blister pack was placed next to them, whereas pink tablets were usually confused with sweets.

Moreover, some cultures seemed to give to colours specific meanings, and this can influence perceptions of medicines as well. For instance, Brieger et al. [34] showed that African respondents clearly associated the colour of a medicine with its effect and purpose. Thus, yellow was accepted as a colour for antimalarial drugs because of perceptions of local symptoms, e.g., eyes turning yellowish during malaria. On the other hand, blue antimalarial drugs showed noncommittal opinions, as this colour had no association with the disease.

### 3.5. Effect of the Colour of Medicines on Acceptability

The remaining 5/18 publications provided anecdotal information about colour in relation to acceptability. Four of these publications were in agreement that the colour of a medicine can affect its appearance and, thus, the willingness of children to take the medicine [31,38,39,44]. For example, Januskaite et al. [44] stated that a “nice” colour of a drug is a contributing factor to a “nice” appearance of a medicine. Moreover, Bryson et al. [38] suggested that dislike of a colour is indicative of a potential aversive response to medications of the same colour, and that it will be a contributing factor in acceptability and ultimately adherence. However, one publication reported that colour seemed to be the least important aesthetic attribute of a medicine according to around 70% of the children in their study [41]. Thus, the authors of such study deemed colour to be a non-relevant attribute affecting acceptability.

### 3.6. Colour and Its Relationship with Flavour

A mention about flavour was reported in 8/18 studies; however, most of them did not assess the relationship between colour and flavour. It is known that children tend to prefer sweet flavours [49,50], and this seemed to be related with specific colours, such as pink and red [51]. Four of the eight studies that mentioned flavour preferences seemed to confirm this when looking at the selections of children’s most liked colours and flavours. In two studies, pink/red were the most liked colours and strawberry was the preferred flavour [35,40]. However, questions around colour and flavour were unrelated to each other. In another study, most of the children believed pink tablets to be sweet compared to the bicoloured or white ones [36]. Moreover, Januskaite et al. [44] reported that when showing yellow 3D printed tablets to the children, their feelings were that they would taste like lemon/orange, again suggesting an association between flavour and colour. Interestingly, the only study that assessed a relationship between colour and flavour of medicines found no apparent association between the two attributes selected by each child [33].

## 4. Discussion

Children tend to like bright colours [18,20,21], and this seems to apply to medicines as well [15,52]. However, it is not clear whether some colours can positively or negatively affect a medicine’s acceptability. Thus, our review aimed to collate information about children’s preferences for colour of oral medicines to understand what is already known from the literature around this topic, and to understand to what extent the addition of a colouring agent can positively or negatively impact acceptability.

Several studies assessing preferences and perceptions for the colour of medicines were performed in the adult population [12,13,14,53,54,55,56], and these studies showed that colour has an impact on drug differentiation, therapeutic effect, and medication adherence. However, to the knowledge of the authors, this is the first review to collect this information in the paediatric population.

The results from this systematic review revealed that colour is an attribute not frequently considered when developing medicines for the children. Only five studies included in this review assessed colour of medicines as a primary aim of their study [29,30,31,33,34], whereas colour was usually mentioned as an anecdotal record.

Results from four studies included in the review suggested that a favourable colour of a medicine seemed to be a contributing factor to a “nice” appearance, and this seemed to be linked with a positive acceptance. However, one large study involving 590 children highlighted that around 70% of the children ranked the colour of a medicine as the least important aesthetic attribute, thus concluding that colour seemed an irrelevant attribute affecting acceptability [41].

Red and pink were the colours most liked by the children for their medicines. This appeared to apply for both solid and liquid dosage forms, although these conclusions were based on few studies, many of which were conducted in the United Kingdom. Surprisingly, other bright colours such as yellow, green, orange, or blue were not frequently selected as favourite colours by the children. For instance, blue and yellow, which were present in all seven studies reporting a colour rating, were never picked as first choices, and yellow was usually one of the least colours selected. This suggests that only specific bright colours seem to be liked by the children for their medicines. Interestingly, the colour white was reported in three studies only, and it was usually in the middle or towards the end of the rating, indicating that this colour was not among those preferred by the children, although frequently applied in pharmaceutical products. Also, brown, black, and green emerged to be unappealing colours for paediatric medicines.

The preference for the colour red is common in the toy and food industries. It was reported that, particularly during early childhood, children are attracted by red toys [18,57], although gender type colour preferences seem to be gradually acquired through increased social contact and school [18]. Moreover, it was reported that a red colour for food or its packaging is perceived sweeter and, thus, preferred compared to blue or green packaging [58].

One possible explanation around children’s preference for red and pink colours in the food and pharmaceutical sector seemed to be their association with sweetness [59], a taste that is generally appreciated by the children [12,60]. Among the studies collected, two publications that looked at children’s preferences for colour and flavour of medicines independently reported that most of the children chose a pink/red colour and strawberry as their preferred flavour [35,40]. Moreover, another study reported that children thought pink tablets to be sweeter compared to the bicoloured and white capsules [36]. However, these studies were all conducted in the United Kingdom or Europe, where strawberry is known to be generally liked by the children [24,61,62]. Children in different countries may show different preferences for different colours and flavours. Interestingly, the only study in the present review that explored a direct relationship between colour and flavour of the medicines selected by the children [33] found no direct association between the two attributes. This highlights challenges of conducting studies of this sort with children, as their responses may vary depending on how the question is formulated or whether any bias was present. Future well-designed studies would be needed to further explore this. Whether colour played an effect on the perceived efficacy of the medication was assessed by two studies only, in 10 to 14-year-old children [37,43]. It revealed that children seemed conscious that colour did not affect a medication’s efficacy. Whether this aspect can be relevant to children and affect their acceptance for medicines in different colours is dubious, particularly in younger children. In adults, several studies investigated colour and its association with a pharmaceutical function [12], or the placebo effect evoked by a colour [12,54,55], or the evaluation of colour associations and their effects on expectations of drugs’ efficacies [56]. Conclusions from all these studies remarked that colour can affect people’s product expectations regarding the efficacy and properties/effects of medicines. However, for children, the meaning evoked by the colour of a medicine appeared to be simpler than in adults. Children may associate the colour with a known flavour, food, or drink, or to the colour of medicines already used, or simply to the colour of something that they like or dislike.

However, the present review was unable to capture the extent to which the colour of a medicine can positively or negatively affect the child’s acceptance of a medicinal product.

Results from this review highlighted a lack of uniformity in the studies conducted around this topic. Colour was usually assessed as an anecdotal record rather than an actual study endpoint, indicating the scarce research conducted around this subject. Studies collected showed a large variability in the methodologies applied to collect information around colour and a frequent lack of complete information around the methodology applied, dosage forms considered, question(s) asked and so on. For instance, none of the studies that included a colour ranking specified the rationale for the colours selected, which differed in number and type from one study to the other.

This review brought to light several gaps in the current knowledge around colour of oral dosage forms in children that would require further consideration. For instance, it was not possible to draw any conclusions around the existence of different colour preferences in paediatric populations of different countries/cultural backgrounds, genders, health status, or age-groups due to the limited number of studies available. Moreover, it was not possible to assess whether differences existed between prescription medicines and OTCs, for which there is more choice and control over choice as well as marketing pressure.

Reasons for this limited research can be the fact that the paediatric pharmaceutical market is not only more difficult to penetrate due to age-related research constraints but also smaller compared to the adult one; thus, less resources are invested around colour preferences for paediatric medicines. Moreover, another key reason is that colouring agents are strictly regulated [7], and their use needs to be justified, thus limiting its research interest.

The legislation governing the use of colouring agents varies from country to country [7]. As stated above, in Europe, the use of colouring agents is regulated by the Directive 2009/35/EC [5]. Moreover, their use in paediatric medicinal products is discouraged by the EMA’s ‘Guideline on pharmaceutical development of medicines for paediatric use’ [22]. The guideline specifies that the use of colouring agents should be discussed and justified in terms of allergenic potential, minimal toxicological implications in the target age group(s), patient acceptability enhancement potential, and role in avoiding accidental dosing errors. If differentiation between similar products is needed, the use of different strategies is preferable [22]. In the United States, colour additives are subject to the FDA approval before they may be used in drugs for both adults and children [63,64], and likewise, this applies to other countries [65].

Restrictions exist because some colouring agents have been associated with hypersensitivity, allergic reactions [66], and potential interactions with some drugs [67]. Thus, the number of colouring agents that are acceptable for use in medicines is limited [68]. Moreover, a colour accepted in one country may not be approved in another, limiting the number of colours that are acceptable for global use.

Thus, it is assumed that to avoid regulatory issues, formulators prefer to not add any colouring agent or to keep the colour used in the adult population, unless strictly necessary, for example, to differentiate between the adult and paediatric strengths or two different strengths of the same product if alternative strategies cannot be applied.

The situation is somehow different for over-the-counter medicines and supplements, where various colours are instead used for marketing reasons. Thus, colour seems to be most widely used to appeal to young consumers. However, not much proprietary information about the rationale behind and justification for the selection of colouring agents for these products is available, although they still need to demonstrate quality, safety, and efficacy.

Results from the present review indicate the need to conduct future patient-centred research on this topic to better understand to what extent colour can affect medicine acceptability in the paediatric population, and if that is the case, what are the factors affecting children’s preferences for coloured medicines? Conducting studies in the targeted population is essential to generate useful knowledge to create medicines that are tailored and accepted by the children, as their preferences may differ from those of adults. A study comparing preferences between healthcare professionals and children about oral formulation characteristics of paediatric medicines showed that, with respect to colour preferences, healthcare professionals selected white, whereas children selected pink as the most preferred colour for paediatric orodispersible tablets [40], suggesting different preferences between the paediatric and adult population and the need for children’s data.

Future studies would be useful to assess if the results observed in this review are generalisable, and whether any difference between cultures, countries, gender, age, health status can be identified. Moreover, it would be interesting to assess whether different types of dosage forms will evoke the same colour preferences and whether any difference in colour preference can be assessed between prescription medicines and over-the-counter products. Finally, it would also be interesting to assess whether matching the colour with a corresponding flavour is important for the children.

As the focus on the acceptability of paediatric medicines is becoming an essential part in the pharmaceutical development, it is foreseen that more attention will be given to the appearance of a medicine in future. The authors would recommend including an assessment of the colour of a dosage form together with other organoleptic characteristics when assessing the acceptability of a dosage form, which may ultimately justify the addition of safe colouring agents into a paediatric medicine.

The main limitation of this systematic review was the identification of relevant papers containing information around colour of medicines in the paediatric population. Colour was rarely studied as a primary outcome, and this led to challenges in defining the search strategy and key words to use for the identification of relevant publications. Colour was hardly reported as a key word or in the title or abstract of the studies, and so, it was necessary to widen the search strategy to make sure relevant papers were identified.

Moreover, studies were not excluded based on their level of methodological quality, but instead, it was decided to keep all the publications containing information about colour, regardless of the risks of bias that this entailed. The reason for this choice was that since colour was usually assessed among the secondary outcomes or reported as anecdotal records, the description of the methodology used was frequently lacking essential detail or lacking at all. Thus, it would not have been possible to assess it. Although there may be a risk that these methodological issues might compromise the interpretation of some data, results were reported as they were presented by the authors without analysing raw data and any form of interpretation was performed.

Finally, the reduced number of publications retrieved, and the fact that they were not always looking at the same aspect around colour, caused an impossibility to draw sound conclusions on several aspects around the perception of colour of medicines in children.

## 5. Conclusions

To the authors’ knowledge, this was the first review summarising information about children’s preferences for colour of oral medicines. Although available data around this topic were scarce, the information collected provided novel insights that could be useful to formulators. The results indicated that children like red and pink medicines over other colours, and this applied for both solid and liquid oral dosage forms. Visual appearance and, thus, the colour seemed to affect children’s expectations for the taste of a medicine, and this can affect adherence. However, many questions remain still unanswered, reinforcing the need for purposefully designed studies in the target population to fill these gaps.

## Figures and Tables

**Table 1:** Scoring system adapted from [28].

Scoring System	Score	Paper Quality
Yes = 1	9–10	High quality
Can’t tell = 0.5	7.5–9	Moderate quality
No = 0	<7.5	Low quality

**Table 2:** Table with summary of studies included in review.

Year & [Ref]	Study Design	Colour Assessed as Primary aim (Yes/NO)	Methodology ^a^	Topic around Colour	Dosage form and Medication Type ^b^	Country	Age (y)	Sample Size	Health Status	Results
1958 [29]	Observational study	Yes	Ten lactose tablets of different colours are showed to the children. Observation of which colours children picked first.	Favourite colour	Tablets NA	United Kingdom	1–8	613	Inpatients and outpatients	Children like bright colours. Colour ranking order: magenta, pink, blue, orange, brown, yellow, white, green, black, and wine.
1996 [30]	Survey Study	Yes	Bottles with liquids of 5 different colours showed to the mothers. They had to indicate their first and second preferences.	Favourite colour	Liquid formulations NA	Papua New Guinea	NA	62 (mothers)	Patients attending health clinic	Red most popular colour, followed by yellow. Blue, brown, and white were not popular.
1996 [31]	Acceptability/ preference questionnaire study	Yes	Acceptability/preference questionnaire.	Effect on acceptability	Liquid suspensions Rx—antibiotics	Arizona, USA	4–12	769	Healthy children	After taking medications, children were asked which had the preferable taste and colour. No mention about the colour of the medicines tested.
1997 [32]	Observational study	No	Not clear how information about colour was collected.	Favourite colour	Granules, dry syrup Rx—antibiotics	Japan	5–8	NA	NA	Children and infants prefer orange and pink. Potential reasons: colours agreeable to children, or children associate them with the fruits’ colours, or colours used in preparations taken before.
2004 [33]	Semi structured interview	Yes	A list of 6 non-medicated liquids in different colours was shown to the children. They had to pick their favourite.	Favourite colour	NA OTC	South Africa	5–6	25	Healthy children	Red was the most popular colour and bubble-gum the favourite flavour. No direct association between colour and flavour observed. Colour ranking order: red, green, white, blue, brown, yellow.
2007 [34]	Focus group discussion	Yes	Open questions about colour preferences/avoidance.	Relationship with efficacy/effect	Tablets Rx—antimalarial	Nigeria, Africa	<5	4 (parents)	NA	Respondents associated medicines and their colour with their effects and purpose. Colour for antimalarial drugs: yellow was accepted, blue not accepted.
2012 [35]	Prospective observational study	No	Open question: “If you had to choose what colour you would like your medicine to be which one would it be?”	Favourite colour	Liquid formulations OTC—analgesic	United Kingdom	5–16	159	Inpatients	Children prefer brightly coloured medicines. Colour ranking order: pink, red, yellow, and blue. Colour is sex dependent: girls significantly prefer pink and boys blue. Preferred flavours: strawberry and banana.
2012 [36]	Questionnaire study	No	Photographed pictures of medicines showed to children.	Relationship with efficacy/effect	Solid dosage forms: tablets and capsules NA	United Kingdom	4–11	182	Healthy children	Majority of children correctly identified the bicolored capsules as medicines compared to the white or pink tablets. Most of the children identified the white tablets as medicines when the blister pack was next to them. Pink tablets less often identified as medicines: 53% of the children identified the pink tablets as sweets compared with just 3% of the bicolored capsules as sweets.
2013 [37]	Cross-sectional survey	No	Close question: “does the colour of medicines affect their action?” Yes—Sometimes—No relation to the colour of medicine.	Relationship with efficacy/effect	NA	Malaysia	11–12	842 (children) 842 (parents)	Healthy children	57.3% (n = 482) of the children think the efficacy of medicines is not related to their colours.
2014 [38]	Interview and questionnaire study used to construct a Medication Adherence Prediction Tool (questionnaire)	No	Open and closed question: “Are there any colours of medicines you like?” Yes/No, “which one(s)?” AND “Are there any colours of medicines you do not like?” Yes/No, “which one(s)?”	Effect on acceptability	NA	United Kingdom	3–11	70	Children with chronic condition	Evidence of the child’s expression of a colour dislike indicative of a potential aversive response to medications of the same colour. This is a contributing factor in acceptability and ultimately adherence.
2015 [39]	Semi-structured face-to-face interviews	No	Anecdotal information about colour reported among the obstacles to medicines administration.	Effect on acceptability	Various (liquids, tablets or capsules, granules, soluble, tablets and melts) NA	United Kingdom	12–18	57 (children) 221 (parents)	Children with chronic condition	An unfavourable colour (“alarming”, off-putting, unappealing, and colourless) associated with 2% (11/542) of medicines prescribed.
2016 [40]	International, multi-site, cross-sectional questionnaire	No	Question not specified. Presumably children had to select their favourite colour from a list of 6 colours.	Favourite colour	Various (liquids, tablets, capsules and ODTs) NA	United Kingdom, Saudi Arabia and Jordan	6–18	104	Healthy children, previous experience taking medications	Pink was the preferred colour for ODTs followed by white, blue, yellow, orange, and purple. Flavour: strawberry was the most preferred, while orange was the least preferred. Gender and age groups showed different colour preferences for ODTs: girls preferred pink ODTs while boys preferred white. Ranking list of flavours: strawberry, orange, cherry, vanilla, mint, lemon, chocolate, and other.
2016 [41]	Age-adapted questionnaire	No	Not specified. Presumably children had to rank aesthetic attributes.	Effect on acceptability	Solid dosage forms: tablets, capsules, chewable tablets, orodispersible tablets, multiparticulates, and mini-tablets NA	United Kingdom and Canada	6–18	590	Healthy children	Colour was ranked as the least important attribute, 70.7% rated it as not important. 48.8% of children showed no specific preference for colour, 25.6% preferred white medicines, and 25.6% preferred coloured medicines.
2019 [42]	Single-centre, prospective crossover experimental study	No	Colour and flavour assessed together. Use of a 5-point hedonic scale to rate each physical 3D tablet. (5 = excellent to 1 = inacceptable).	Favourite colour	3D printed 3D printed tablet RX—metabolic disease	Spain	3–16	4	Patients with chronic condition	Six types of formulations tested (6 flavour/colour combinations): strawberry-red, orange-orange, lemon-yellow, raspberry-light blue, banana-light green, and coconut-black. Preferred combination (but not statistically significant): orange-orange. Worst rated combination: coconut-black.
2019 [43]	Cross-sectional questionnaire	No	Closed question: “Medicine colour (coloured or white) affects medicine drug efficacy?” Yes—No—I don’t know.	Relationship with efficacy/effect	NA NA	Indonesia	10–14	503	Healthy children	Medicine colour (coloured or white) affects a drug efficacy: yes 22.5%, no 38.2%, don’t know 39.4%.
2020 [44]	A visual preference semi-structured survey and a short electronic questionnaire	No	Anecdotal records about colour collected from an open question where participants could add their comments.	Effect on acceptability	3D printed tablets NA	UK	4–11	368	Healthy children	The majority stated that the 3D printed tablets had a ‘nice colour’. Appearance was closely followed by perceived taste: the 3D printed tablets looked like a gummy/sweet or that they would taste like a lemon/orange. Children show higher preference towards brightly coloured medicines.
2022 [45]	Pre-formulation study with survey about children’s preferences for taste and colour	No	Survey to determine flavour and colour preferences. List of 7 colours used.	Favourite colour	Hydrogel vehicle to improve oral administration of solid dosage forms NA	Portugal	<12	157	Healthy children	Colours more selected: red and pink. Blue was also chosen as one of the favourite colours. Children less likely to choose brown, green, orange, and yellow. Most girls chose pink, most boys preferred green medicines. Strong liking for sweet flavours. Flavour ranking: strawberry, vanilla, caramel, grape, banana, mint, and orange.
2022 [46]	Prospective observational study	No	Colour was assessed indirectly in a questionnaire, and it was one of the characteristics listed that participants had to rank.	Favourite colour	Liquid and solid NA	Germany	6–17	103	Patients with chronic condition	Red colour was the most mentioned by both parents and children while describing their ideal medicine: ‘bright red’ tablet (child), pink or red liquid (parent), colourful (parent).

^a^ Methodology used to assess children’s preferences/opinions for the colour of medicines. ^b^ Type of medication: Over-the-counter (OTC) or prescription medicine (Rx), and drug classes.

**Table 3:** Geographical locations of the studies included in the review; some studies were conducted in more than one country.

No. of Studies	Country	Continent
1	Germany	Europe
1	Portugal	
1	Spain	
8	United Kingdom	
1	Indonesia	Asia
1	Japan	
1	Jordan	
1	Malaysia	
1	Saudi Arabia	
1	Canada	North America
1	United States	
1	Nigeria	Africa
1	South Africa	
1	Papua New Guinea	Oceania

**Table 4:** Methodology used in each study to collect information about colour of medicines.

Methodology	No. of Studies	Ref
Evaluation of coloured medicines or photographed medicines	4	[29,30,33,36]
Hedonic scales/acceptability preference questionnaires	2	[31,47]
Open or closed questions or mix of the two	5	[34,35,37,38,43]
List of colours to choose from	1	[45]
Information not available	6	[32,39,41,44,46,48]

## Data Availability

No new data were created or analysed in this study. Data sharing is not applicable to this article.

## Appendix A

**Table A1:** Search strategy used in each database and number of publications retrieved.

Database	Search Word	Results
PubMed	Search: (((colour OR color) AND (medicine *[Title/Abstract] OR dosage form *[Title/Abstract] OR drug *[Title/Abstract])) AND (child * [Title/Abstract] OR paediatric[Title/Abstract] OR pediatric * [Title/Abstract])) AND (accept * [Title/Abstract] OR perception[Title/Abstract] OR preference[Title/Abstract] OR favourite[Title/Abstract] OR compliance[Title/Abstract] OR adherence[Title/Abstract])	57
Scopus	(ALL (colour OR color) AND TITLE-ABS-KEY (medicin * OR “dosage form” OR drug) AND TITLE-ABS-KEY (child OR paediatric OR pediatric *) AND TITLE-ABS-KEY (accept * OR perception OR preference OR favourite OR compliance OR adheren *))	712
MEDLINE (Ovid)	(colour or color).mp. [mp = title, book title, abstract, original title, name of substance word, subject heading word, floating sub-heading word, keyword heading word, organism supplementary concept word, protocol supplementary concept word, rare disease supplementary concept word, unique identifier, synonyms] (child or paediatric or pediatric).mp. [mp = title, book title, abstract, original title, name of substance word, subject heading word, floating sub-heading word, keyword heading word, organism supplementary concept word, protocol supplementary concept word, rare disease supplementary concept word, unique identifier, synonyms] (medicin* or dosage form or drug).mp. [mp = title, book title, abstract, original title, name of substance word, subject heading word, floating sub-heading word, keyword heading word, organism supplementary concept word, protocol supplementary concept word, rare disease supplementary concept word, unique identifier, synonyms] (accept* or perception or preference or favourite or favorite or compliance or adheren*).mp. [mp = title, book title, abstract, original title, name of substance word, subject heading word, floating sub-heading word, keyword heading word, organism supplementary concept word, protocol supplementary concept word, rare disease supplementary concept word, unique identifier, synonyms] 1 and 2 and 3 and 4	140
Web of Science	All fields—colour OR color AND topic—child OR paediatric OR pediatric* AND topic—medicin* OR dosage form OR drug AND topic—accept* OR perception OR preference OR favourite OR favorite OR compliance OR adheren*	80
Total studies identified		989
